# From isolation to implantation: a concise review of mesenchymal stem cell therapy in bone fracture repair

**DOI:** 10.1186/scrt439

**Published:** 2014-04-15

**Authors:** Luke Watson, Stephen J Elliman, Cynthia M Coleman

**Affiliations:** 1 Orbsen Therapeutics Ltd, University Road, Galway City, Ireland; 2 National University of Ireland Galway, University Road, Galway, Ireland

## Abstract

Compromised bone-regenerating capability following a long bone fracture is often the result of reduced host bone marrow (BM) progenitor cell numbers and efficacy. Without surgical intervention, these malunions result in mobility restrictions, deformities, and disability. The clinical application of BM-derived mesenchymal stem cells (MSCs) is a feasible, minimally invasive therapeutic option to treat non-union fractures. This review focuses on novel, newly identified cell surface markers in both the mouse and human enabling the isolation and purification of osteogenic progenitor cells as well as their direct and indirect contributions to fracture repair upon administration. Furthermore, clinical success to date is summarized with commentary on autologous versus allogeneic cell sources and the methodology of cell administration. Given our clinical success to date in combination with recent advances in the identification, isolation, and mechanism of action of MSCs, there is a significant opportunity to develop improved technologies for defining therapeutic MSCs and potential to critically inform future clinical strategies for MSC-based bone regeneration.

## Introduction

Of the millions of long bone fractures incurred annually, approximately 10% result in impaired healing or non-union. Non-union fractures form as the result of significant trauma or a metabolic disease in which injury to the bone becomes atrophic rather than regenerating. This compromised bone-regenerating capability is a complication associated with reduced bone marrow (BM) progenitor cell numbers compounded by suppressed progenitor cell proliferation as a result of the disease itself [1]. Without surgical intervention, malunions result in severe lifestyle impairment, immobilization, and disability. Standard clinical methods for encouraging bone repair include distraction osteogenesis, autologous bone grafting, allograft demineralized bone matrix (DBM), and bone graft substitutes. However, these interventions are invasive and laden with hardware and result in donor site morbidity.

BM-derived mesenchymal stem cells (MSCs) represent an attractive therapeutic to stimulate the repair of a long bone fracture with their expansion potential, osteogenic capability, and potential to home to a site of injury [2]. Systemically administered MSCs localize in the lungs and liver within 24 hours of administration [3] and in the spleen, brain, or skin for the following 1 to 2 months [4], but when delivered in the presence of a long bone fracture, the circulating MSCs home to the fracture site [3] and integrate into the host marrow, bone, and cartilage [4].

MSC contribution to bone repair has been well established in trauma and disease-based pre-clinical models. Bruder and colleagues [5] first demonstrated that the implantation of a scaffold supplemented with BM MSCs supported osteogenesis over an empty scaffold, including the formation of a reparative callus that was absent in defects treated with scaffold alone. Furthermore, implanting pre-differentiated osteogenic MSC-scaffold constructs resulted in superior healing over scaffold alone [6]. Perhaps more importantly, the administration of MSCs with DBM into a clinically relevant model of diabetes resulted in augmentation of fracture healing over those that did not receive MSCs [7]. For additional information on the delivery of MSCs in combination with matrices for orthopedic repair, including the viability and retention of the transplanted cell, please refer to Bulman and colleagues [8].

Contrasting results demonstrate that autografting is more beneficial than defects treated with scaffold alone or cell-seeded scaffolds. Berner and colleagues [9] demonstrated comparable results in autologous MSC-seeded scaffolds, allogeneic MSC-seeded scaffolds, and empty scaffolds in terms of biomechanics; however, a greater bone volume and torsional stiffness were identified in autograft-treated defects. Bensaïd and colleagues [10] similarly observed superiority of autograft in healing critical-sized tibial defects over MSC-seeded hydroxyapatite (HA) constructs, whereas Cipitria and colleagues [11] reported a similar biomechanical stability and mineral density in MSC-seeded scaffolds as compared with autograft; however, there was reduced bone volume in the autograft-treated groups. Regardless, these studies clearly identify BM-derived MSCs as a viable, minimally invasive therapeutic option to treat fractures without the added complication of donor site morbidity associated with autograft.

## Mesenchymal stem cells: recent advances in fundamental stromal cell biology

The concept of MSCs as progenitor cells was initially based on the identification of clonogenic fibroblastic cells within the BM that could differentiate into adipocytes, osteoblasts, and chondrocytes *in vitro*[12]. In the mid-1990s, BM-derived MSC preparations rapidly entered clinical testing to either replace cells in damaged skeletal tissues and, more recently, to provide paracrine signals to repair vascular injury or modulate pathological immune responses [13]. Progress has been made in elucidating the identity, location, heterogeneity, and physiological functions of the stromal cell populations in BM that expand in culture to generate MSCs. However, the mechanisms by which MSCs contribute to the homeostasis of tissues remains poorly understood.

The hypothesis that MSCs represent *bona fide* skeletal progenitor cells that generate skeletal tissues *in vivo* has been validated by several recent studies. First, with the serial transplantation of clonal CD45^−^CD146^+^ MSCs from human BM, Sacchetti and colleagues [14] demonstrated that cloned human MSCs retain the capacity to generate an ectopic bone and marrow compartment (hematopoietic microenvironment; HME) upon serial transplantation *in vivo*. Similar MSCs can be prospectively isolated from mouse marrow and enzyme-dissociated bone by using antibodies to platelet-derived growth factor receptor alpha (PDGFRα; CD140a) and stem cell antigen Sca-1 [15]. As with the CD146^+^ MSC isolated by Sacchetti and colleagues [14], the PDGFRα^+^Sca-1^+^ MSCs are capable of re-populating the HME when transferred to a donor.

Using the *CXCL12* promoter to drive diphtheria toxin receptor (DTR), Omatsu and colleagues [16] used diphtheria toxin to ablate CXCL12-expressing stromal cells *in vivo* by demonstrating that mouse perivascular marrow stromal cells expressing CXCL12 were necessary for hematopoietic stem cell (HSC) proliferation and served as progenitors for osteoblasts and adipocytes. At the same time, the Frenette laboratory [17] reported that similar perivascular marrow stromal cells express the intermediate filament, nestin. Using a *nestin*-enhanced green fluorescent protein (eGFP) transgenic, they showed that nestin-eGFP^+^ CD45^−^ cells express CXCL12, localize near HSCs, and retain the majority of the colony-forming unit-fibroblasts content of mouse BM. Lineage tracing of CD45^−^nestin^+^ BM cells reveal contribution of the MSCs to the long bone and growth plate cartilage [17]. Greenbaum and colleagues [18] further selectively deleted CXCL12 expression from distinct stromal cell populations, including osteoblasts, endothelial cells, and primitive stromal cells. This study indicated the presence of a distinct MSC population expressing the transcription factor PRX1 (*Prrx1*), supporting different aspects of hematopoiesis. The identified PRX1^+^CXCL12^+^ MSCs did not express nestin or leptin receptor, suggesting that PRX1^+^ MSCs may represent a precursor to cell populations previously identified.

The Scadden laboratory identified marrow stromal cells responsible for bone regeneration by using myxovirus resistance-1 (*Mx1*) promoter-specific lineage tracing [19]. Unlike the nestin^+^ and CXCL12^+^ MSCs, Mx1^+^ contributed to the osteogenic lineages but not adipogenic or chondrocytic lineages. They demonstrated that an ‘MSC-like’ cell population expressing the transcription factor Mx1 serves as a progenitor for osteoblasts following fracture and demonstrates characteristics of MSCs and *bona fide* stem cells in serial transplantation experiments.

Finally, two groups took different approaches to ablate fibroblast activation protein-alpha (FAP^+^) stromal cells from the tumor microenvironment. The Fearon laboratory used the same DTR/DTX system driven by the *fap* promoter to ablate FAP^+^ stromal cells from colon and pancreatic tumors. Notably, the ablation of FAP^+^ cells in healthy animals caused muscle wasting and anemia in the marrow, associated with deregulated hematopoiesis [20]. At the same time, the Rosenberg laboratory used chimeric antigen receptor (CAR) T-lymphocytes design to target FAP^+^ stroma with several murine tumors. Consistent with the Fearon report, Tran and colleagues [21] observed cachexia and osteopenia in mice treated with the FAP-targeting CAR T cells and found that these phenotypes were associated with a loss of CD45^−^PDGFRα^+^Sca1^+^ stromal cells in the BM.

Although there are still outstanding questions regarding the overlap of cells expressing FAP, nestin, CXCL12, Mx1, PRX1, and leptin receptor, these studies have yielded new knowledge regarding the identification *in vivo* role of MSCs, in particular their functional roles in maintaining bone regeneration, marrow, and muscle homeostasis. This recent evidence indicates that cells which serve as precursors to culture-expanded MSCs arise from a spectrum of *bona fide* progenitor cells that can be prospectively identified by their expression of novel functional proteins.

Equally important work has been conducted in human cell and tissue samples. Several groups have recently reported results of fluorescence-activated cell sorting-based purification of stromal cell populations from human BM by using multiple cell surface markers, including CD271, CD56, Stro-3, MSCA-1, and CD146 [22]. These studies confirm the presence of distinct stromal cell populations which differ in their differentiation capacities as well as in their relative abundance during fetal development and aging. Tormin and colleagues [23] demonstrated that these functional differences may correlate with differences in anatomical location as CD271^+^CD146^+^ cells were found in perivascular regions and CD271^+^CD146^−^ cells at endosteal surfaces. In addition, Crisan and colleagues [24] demonstrated that stromal cells expressing the pericyte markers CD146 and NG2 are confined to perivascular regions and can be identified by cytometry from several human tissues, including skeletal muscle, pancreas, fat, placenta, and BM. Although both human and murine studies highlight the heterogeneity of stromal cell populations, they also highlight the lack of specific markers that enable prospective isolation of equivalent MSCs from experimental murine species and humans. Defined homogeneous cells may elicit greater therapeutic activity than heterogeneous MSC populations. In 2007, Sacchetti and colleagues [14] demonstrated that creation of the HME could be achieved only by adoptive transfer of defined CD146^+^ human MSCs; this HME was not produced by heterogeneous MSCs. Similarly, adoptive co-transfer of either mouse ALCAM^+^/Sca1^−^ MSCs [25] or human CD146^+^ MSCs [26] enhance the engraftment and activity of co-transplanted HSCs when compared with heterogeneous MSCs. With regard to clinical development, these advances in fundamental stromal cell biology provide a better understanding of stromal/perivascular cell heterogeneity and function within the BM and other tissues. This knowledge will inevitably lead to improved technologies for defining therapeutic MSCs at the point of isolation and has the potential to critically inform future clinical strategies for MSC-based bone regeneration.

## Mesenchymal stem cell mechanism of action

The method by which MSCs home to the site of injury is widely debated, but chemoattractant molecules released at the site of bone injury draw MSCs to participate in the healing process. At least 19 chemokine receptors are expressed by MSCs with varying significance to the chemotactic process [27]. Expression of stromal cell-derived factor 1 (SDF-1) by cells of the stromal niche is a well-documented attractant for circulating CXCR-4-expressing HSCs [28]. Mounting evidence suggested that the SDF-1/CXCR4 complex played a similar role in the attraction of MSCs to the site of trauma via an upregulation of CXCR4 by MSCs of the injured host [29]. Kitaori and colleagues [30] proposed SDF-1 as the primary chemoattractant present in the periosteum of a murine bone grafting model promoting host-progenitor cell recruitment and activation, whereby administration of an anti-SDF-1 neutralizing antibody significantly inhibited new bone formation. A range of other factors have been documented as chemotactic for MSCs, including RANTES, MIP-1α, MCP-1, CCL25, and CXCL16, but the mechanism of systemically administered MSCs homing to an injury is likely attractant/receptor-based [31].

### Direct mesenchymal stem cell differentiation

In healthy repair, host progenitors contribute to fracture repair by migrating to the repair callus, where they contribute directly to remodeling by differentiation into osteoblasts [32]. In a poorly repairing fracture due to disease, administered MSCs must compensate for the malfunctioning host progenitors. Pereira and colleagues [33] showed that MSCs expanded in culture and injected into irradiated mice act as precursors for bone and cartilage for at least 5 months. Subsequently, BM administration to a non-ablated mouse model demonstrated the engraftment of MSCs, including their osteocytic differentiation [34]. Therefore, transplanted MSCs have the potential to contribute directly to repair by differentiation.

In mice, systemically administered bioluminescent MSCs migrate to the site of a fracture and integrate into the callus, contributing to repair by secreting bone morphogenetic protein (BMP)-2. Transplanted MSCs localized in the margins of the woven bone and differentiated into osteoblasts, as confirmed by their expression of osteocalcin [35]. In humans, the osteogenic capacity of transplanted BM, and therefore MSCs, has been demonstrated in a small-scale clinical study in children with osteogenesis imperfecta. Functional engraftment of these systemically administered allogeneic MSCs was observed 100 days after administration with associated clinical improvements [36]. Systemic administration can therefore result in homing of MSCs and their direct contribution to orthopedic repair.

The addition of osteo- or angiogenic growth factors to engineer tissue repair *in situ* has gained popularity. MSC differentiation by the addition of growth factors such as BMP-2 or -7 results in superior osteoblastic differentiation and increased mechanical integrity when applied *in vivo*[3][37,38]. Furthermore, the combination of vascular endothelial growth factor and BMPs simulates both osteogenesis and angiogenesis, resulting in robust bone growth and increased mechanical strength [3]. However, Tortelli and colleagues [39] and Scotti and colleagues [40] have demonstrated that fracture repair is dependent on the maturation state of the implanted cell. Transplanted osteoblastic progenitors stimulate host cells to repair the fracture via intramembranous ossification while undifferentiated or chondrogenically primed MSCs instruct the host to repair via endochondral ossification (EO), a process reminiscent of embryonic skeletal development. The presence of the chondrogenic MSC-recruited host CD31^+^ endothelial cells increased vascularity and consequently host osteoprogenitor cell recruitment, enabling the development of high-quality reparative tissue through EO. Scotti and colleagues [41] have further presented promising results by using ‘developmental engineering’ to recapitulate EO with human MSC-seeded collagen scaffolds implanted in a mouse, resulting in complete formation of a bone organ.

### Indirect paracrine influence

The administration of MSCs to a site of injury or in a disease state will expose the cells to an environment not conductive to cell survival. However, recent findings of von Bahr and colleagues [42] have demonstrated that the therapeutic efficacy of these cells did not depend on their sustained viability, but instead on their indirect paracrine influence. Fractures trigger the secretion of a variety of proinflammatory cytokines, including IL-1α, IL-1β, IL-6, and IL-18 and TNF-α [43]. This controlled inflammatory reaction establishes the angiogenic and osteogenic environment that facilitates repair. Prolonged or chronic inflammation, as found in disease states, is inhibitory to bone repair and contributes to non-union formation. In healthy repair, TNF-α recruits osteoclasts and MSCs to the fracture site while causing apoptosis of hypertrophic chondrocytes in support of EO in the early inflammatory response [44]; however, prolonged elevated TNF-α levels inhibit repair by accelerating cartilage removal and elevating osteoclast numbers [45].

The immune conditioning properties of MSCs offer a unique opportunity to regulate the extreme immune response during abnormal fracture repair. As demonstrated in human clinical trials, MSCs administered for the treatment of graft-versus-host disease result in dramatically improved survival rates [46]. Granero-Moltó and colleagues [35] further demonstrated MSC modulation of the local inflammatory environment at a fracture site and this led to lower IL-1β, IL-6, and TNF-α expression in the repair callus and thus to increased biomechanical stability. Therefore, systemically administered MSCs are efficacious in regulating exaggerated immune responses and thereby can contribute indirectly to fracture repair.

Transplanted MSCs also instruct the resident host progenitors, ensuring their optimal contribution to repair in addition to modulating the immune milieu at the fracture site. Xenotransplantation studies demonstrated the instructive capability of donor MSCs on host progenitors when quail progenitors were implanted subcutaneously into mice. In the first month of repair, donor progenitors were responsible for the neogenic osseous tissue; however, with time, the host cells were predominately responsible for the heterogeneous donor-host bone [47]. More recently, MSC-seeded ceramic scaffolds were shown to be depleted of the transplanted MSCs and replaced by the host’s own circulating osteoprogenitors *in vivo*. A wave of infiltrating host endothelial cells was observed to respond to the donor MSCs, followed by an influx of CD146^+^ pericytes. Together, the transplanted MSCs condition the local environment to stimulate host angiogenesis and therefore osseous repair [48]. Still, the contribution made by exogenously implanted MSCs in fracture repair remains unclear, but the potential for MSCs as an orthopedic cell therapy strategy is promising.

## Clinical application of mesenchymal stem cells

In 1995, the first MSC clinical study used autologous, culture-expanded MSCs in patients with hematological malignancies, demonstrating safety with no reports of adverse events [49]. Since this first trial, over 3,000 patients enrolled in over 100 clinical trials have met safety endpoints with no serious adverse events reported to date.

In 2001, a correspondence to the *New England Journal of Medicine*[50] presented the first preliminary clinical data (a pilot, unblended, uncontrolled, unrandomized study) on three patients who received cell-based treatment for long bone non-unions due to trauma. Here, autologous BM was harvested and the MSCs culture expanded before implantation in combination with an HA scaffold. At 15 to 27 months, all patients had promising callus formation and the absence of complications [50]. After 6 to 7 years, complete fusion was observed, including integration with the healthy host bone, thereby demonstrating the therapeutic potential of MSCs on an osteoconductive scaffold [51]. Furthermore, osteogenically pre-differentiated patient-specific autologous MSCs clinically implanted on HA demonstrated ‘healing potential’ supported by incorporation with the host bone, increased radiographic density, and no loosening of the implants upon follow-up [52]. More recently, Liebergall and colleagues [53] clinically demonstrated that administration of MSCs in combination with DBM directly administered to tibial fractures resulted in more rapid fracture union, whereas Giannotti and colleagues [54] have demonstrated complete healing of all patients through the application of autologous cultured MSCs, which were osteogenically differentiated and then implanted into the fracture in autologous fibrin scaffolds. Together, these preliminary studies demonstrate the safety and potential efficacy of autologous MSCs in orthopedic repair.

Although recent pre-clinical reports have raised the concern that implanted BM-derived MSCs may stimulate harmful ectopic bone formation by the host [55], the published clinical reports discussed above have fully examined the site of MSC administration radiographically and no evidence of ectopic bone formation has been described. Furthermore, von Bahr and colleagues [42], who examined autopsy material of 18 patients who received MSC transplantations, have found no evidence of sustained retention of the transplanted cells or their involvement in forming ectopic tissue.

Future clinical application of MSCs to support long bone fracture healing will mature from the elementary (the implantation of heterogeneous autologous BM combined with a scaffold) to the advanced systemic application of a selected allogenic osteoprogenitor. The transition from autologous therapy to allogeneic treatment is a prerequisite, as those who require progenitor cell therapy to heal a fracture often have reduced progenitor cell number themselves, the initial reason for their development of a non-union [56]. Unfortunately, all current clinical investigations, summarized in Table 1, are fully focused on autologous heterogenic cell-based therapy. Most interestingly, though, is the significant transition of nearly half of the ongoing studies to the minimally invasive, local, or systemic administration of MSCs without the addition of a carrier.

**Table 1:** Summary of ongoing clinical investigations involving bone marrow-derived mesenchymal stem cells applied to long bone fracture

**Identifier**	**Location**	**Date submitted**	**Treatment**	**Number of patients**
NCT00250302	Israel	Nov. 2005	Autologous bone marrow-derived MSCs loaded onto a carrier and implanted into the fractured tibia	24
NCT00512434	France	Aug. 2007	Concentrated autologous bone marrow delivered percutaneously to a tibial non-union	85
NCT00557635	France	Nov. 2007	Osseous matrix with concentrated autologous marrow to treat tibial or femoral pseudo-arthrosis	50
NCT01206179	Iran	Sept. 2010	Percutaneous MSC injection into the fracture callus of a long bone	6
NCT01429012	Belgium	Sept. 2011	Autologous bone marrow-derived MSCs percutaneously administered to atrophic non-union	40
NCT01435434	Israel	Sept. 2011	Tibia or femur non-union treated with DBM and autologous ‘buffy coat’ progenitors	Not Provided
NCT01581892	Spain	April 2012	Ficoll isolated autologous MSCs mixed with osteogenic matrix and implanted at the site of long bone non-union	30
NCT01626625	Indonesia	June 2012	Expanded bone marrow-derived MSCs applied to non-unions with HA as compared with autograft	10
NCT01725698	Indonesia	Nov. 2012	Osteoconductive matrix with MSCs and BMP-2 to treat critical-sized bone defects	5
NCT01788059	Iran	Jan. 2013	Autologous bone marrow MSCs isolated by Ficoll gradient, then implanted into tibial non-unions	18
NCT01842477	France	April 2013	Autologous bone marrow cells expanded and implanted with scaffold at the site of non-union in a femur or tibia	30

This table includes active investigations listed on http://www.clinicaltrials.gov as of 1 November 2013. BMP, bone morphogenetic protein; DBM, demineralized bone matrix; HA, hydroxyapatite; MSC, mesenchymal stem cell.

## Conclusions

Compounded with recent advancements in the identification of osteogenic precursors, the good manufacturing practice-grade isolation of clinically efficacious cells is an attractive possibility, enabling the delivery of a smaller dose of cells to the patient. The challenge for the upcoming decade of orthopedic research is therefore one of translation: to render, from the outstanding recent advancements in progenitor cell identification and our improved understanding of the therapeutic mechanism of action, clinically relevant therapies to advance the treatment of long bone non-union.

## Abbreviations

BM: Bone marrow; BMP: Bone morphogenetic protein; CAR: Chimeric antigen receptor; DBM: Demineralized bone matrix; DTR: Diptheria toxin receptor; eGFP: Enhanced green fluorescent protein; EO: Endochondral ossification; FAP: Fibroblast activation protein alpha; HA: Hydroxyapatite; HME: Hematopoietic microenvironment; HSC: Hematopoietic stem cell; IL: Interleukin; MSC: Mesenchymal stem cell; Mx1: Myxovirus resistance-1; PDGFRα: Platelet-derived growth factor receptor alpha; Sca: Stem cell antigen; SDF-1: Stromal cell-derived factor-1; TNF-α: Tumor necrosis factor-alpha.

## Competing interests

The authors declare that they have no competing interests.
